# HOXA10 induces BCL2 expression, inhibits apoptosis, and promotes cell proliferation in gastric cancer

**DOI:** 10.1002/cam4.2440

**Published:** 2019-07-30

**Authors:** Chenlong Song, Yang Han, Huan Luo, Zhiwei Qin, Zhengqian Chen, Yuan Liu, Su Lu, Huimin Sun, Chongzhi Zhou

**Affiliations:** ^1^ Department of General Surgery, Shanghai General Hospital Shanghai Jiao Tong University School of Medicine Shanghai China; ^2^ Department of Gastric Surgery Fudan University Shanghai Cancer Center Shanghai China; ^3^ Department of Oncology, Shanghai Medical College Fudan University Shanghai China; ^4^ Department of Neurology The Fifth Affiliated Hospital of Zheng Zhou University Zhengzhou China; ^5^ Department of Pathology, Shanghai General Hospital Shanghai Jiao Tong University School of Medicine Shanghai China

**Keywords:** apoptosis, BCL2, gastric cancer, HOXA10, proliferation

## Abstract

Homeobox A10 (HOXA10) has been implicated critical for the promotion of carcinogenesis, but the underlying mechanism between HOXA10 and malignant gastric cancer (GC) phenotype remains elusive. In the present study, we analyzed and validated that HOXA10 and BCL2 expressions were elevated both at the mRNA and protein levels in GC tissues. Upregulated HOXA10 promoted GC cell proliferation with reduced apoptosis in vitro and accelerated GC tumor growth in vivo. Bioinformatics analysis and quantitative real‐time polymerase chain reaction (qRT‐PCR) experiment inferred that HOXA10 might upregulate the expression of BCL2. By performing western blot, chromatin immunoprecipitation and quantitative PCR (ChIP‐qPCR), and rescue experiment, we found that HOXA10 might bind to BCL2 promoter region, induce its expression, and thus inhibit intrinsic apoptosis pathway. Moreover, higher expression of HOXA10 and BCL2 predicted poor overall survival (OS) in GC patients. In summary, our study indicated that HOXA10 was upregulated in GC, and that HOXA10 might promote cell proliferation by elevating BCL2 expression and inhibiting apoptosis.

## Graphical abstract

In summary, we demonstrated that expression of HOXA10 and BCL2 was significantly upregulated in gastric cancer (GC) tissues. HOXA10 overexpression promoted GC cell proliferation, enhanced tumor growth, and inhibited apoptosis. Through bioinformatics analysis, western blot, chromatin immunoprecipitation and quantitative PCR (ChIP‐qPCR), and the rescue experiment, we found that HOXA10 might upregulate the expression of BCL2 via binding to the BCL2 promoter region. All these findings suggested that HOXA10 might promote GC cell proliferation by upregulating BCL2 expression and inhibiting apoptosis.

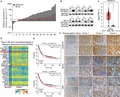

## 1 INTRODUCTION

Gastric cancer (GC) is one of the most common and fatal malignancies worldwide.^1^ Although significant progress has been achieved in surgery, chemotherapy, and radiotherapy for GC in recent years, GC remains a serious health problem with a low 5‐year overall survival (OS) rate and poor prognosis. This may be attributable in part to insensitivity and resistance to chemotherapy.^2^ Therefore, a more thorough understanding of the molecular mechanisms that drive gastric carcinogenesis and identification of new useful biomarkers as well as therapeutic strategies are urgently needed.

Homeobox or HOX genes encode conserved transcription factors that regulate anterior‐posterior (A‐P) developing pattern and have been elaborated in detail over the last decade for their roles in the mechanisms underlying embryonic development.^3^ Recently, increasing evidence indicates that dysregulation of HOX family members maybe appear critical for the promotion of carcinogenesis.^4^, ^5^, ^6^ Preciously, we performed cDNA microarray and validated several differentially expressed HOX genes in GC—especially HOXA1, HOXA10, and HOXA13—with significantly higher expression in the cancerous tissues.^7^ Intensive investigation on HOXA1 suggested that it accelerated cell proliferation partly via cyclin D1.^8^ Studies on HOXA10 indicated that upregulation of HOXA10 in GC encouraged cell growth and was associated with worse clinical outcome.^9^

However, the functional role and underlying molecular mechanism between HOXA10 and cell proliferation in GC have not been fully elaborated. This prompted us to focus on the regulatory role of HOXA10 on cell proliferation and explore conceivable mechanisms in GC.

Apoptosis, a form of programmed cell death, is pivotal for suppressing tumor growth and guaranteeing healthy tissue homeostasis.^10^, ^11^ Targeting apoptosis pathways to induce cancer cell death has always been a high‐priority strategy for cancer therapy.^12^, ^13^ The intrinsic (mitochondrial) apoptosis pathway is regulated by the BCL2 family members, which are usually dysregulated in cancers.^10^, ^14^, ^15^

In the study reported here, we found that HOXA10 was upregulated in GC tissues both at the mRNA and protein levels. A series of in vitro and in vivo experiments indicated that overexpression of HOXA10 in GC cells accelerated cell proliferation and tumor growth with reduced apoptosis. Elevated HOXA10 upregulated BCL2 expression and thus inhibited intrinsic apoptosis pathway with downregulated expression of cleaved forms of Caspase‐9, Caspase‐3, and PARP. Moreover, bioinformatics analysis, chromatin immunoprecipitation and quantitative PCR (ChIP‐qPCR), and rescue experiment revealed that HOXA10 might regulate BCL2 expression via binding to its promoter region. These results provided evidence that HOXA10 might regulate BCL2 apoptosis signaling pathway and promote GC cell proliferation, which may contribute to finding new treatment strategies for GC.

## 2 MATERIALS AND METHODS

### 2.1 Patients and specimens

Fresh GC tissues and adjacent normal tissues for quantitative real‐time polymerase chain reaction (qRT‐PCR) were collected from 50 GC patients in the Department of General Surgery of Shanghai General Hospital. All the patients provided written informed consent. This study was approved by the Ethics Committee of Shanghai General Hospital.

### 2.2 RNA extraction and qRT‐PCR analysis

Total RNA from GC cells and tissue specimens was extracted with Simply P Total RNA extraction Kit (BioFlux), and 2 μg of total RNA from each sample was reversely transcribed into cDNA using PrimeScript^™^ RT Master Mix (Perfect Real Time; Takara). qRT‐PCR was performed using SYBR^®^ Premix Ex Taq^™^ (Tli RNaseH Plus; Takara) in a Light Cycler Real‐time PCR System (Roche). The amplification was performed as follows: an initial denaturation step for 30 seconds at 95°C followed by 40 cycles of denaturation for 5 seconds at 95°C, annealing and extension for 30 seconds at 60°C. The specific primers used were as follows: GAPDH‐F: GGGAAGGTGAAGGTCGGAGT; GAPDH‐R: GGGGTCATTGATGGCAACA; HOXA10‐F: TCACGGCAAAGAGTGGTC; HOXA10‐R: AGTTTCATCCTGCGGTTCTG; BCL2‐F: ACTGGCTCTGTCTGAGTAAG; BCL2‐R: CCTGATGCTCTGGGTAAC. GAPDH was used as an internal control, and the relative quantities (Δ cycle threshold values) of each transcript were normalized to GAPDH. Each reaction was repeated in triplicate. The fold changes (2^−ΔΔCt^) in HOXA10 and BCL2 mRNA expression were calculated using the following formulae: HOXA10ΔCt = (Avg. HOXA10_Ct − Avg. GADPH_Ct), HOXA10ΔΔCt = (HOXA10ΔCt_tumor − HOXA10ΔCt_non‐tumor); BCL2ΔCt = (Avg. BCL2_Ct − Avg. GADPH_Ct), BCL2ΔΔCt = (BCL2ΔCt_tumor − BCL2ΔCt_non‐tumor).

### 2.3 Protein extraction and western blot analysis

Total protein was isolated using RIPA Lysis Buffer (Beyotime Biotechnology) and protein concentration was measured with a BCA protein assay kit (Beyotime Biotechnology). Forty microgram of protein was separated by sodium dodecyl sulfate‐polyacrylamide gel electrophoresis and then transferred onto the polyvinylidene fluoride (PVDF) membranes. The membranes were blocked in 5% fat‐free milk solution containing 0.1% Tween‐20 for 1 hour at room temperature. Later, membranes were incubated at 4°C overnight with primary antibodies against HOXA10 (1:1000; GeneTex), GAPDH (1:5000; proteintech), and BCL2, Bax, cleaved Caspase‐9, cleaved Caspase‐3, and cleaved PARP (1:1000; Cell Signaling Technology). Subsequently, the membranes were incubated with HRP (horseradish peroxidase)‐conjugated secondary antibodies (1:10 000; Jackson ImmunoResearch Inc) for 1 hour at room temperature. After washing with TBST buffer, the targeted proteins were visualized with Immobilon^™^ Western Chemiluminescent HRP Substrate (Millipore).

### 2.4 Lentiviral shRNA‐mediated knockdown and HOXA10 overexpression

Lentiviruses targeting human HOXA10 gene were obtained from GeneChem (Shanghai, China). The viruses were used to infect cells in the presence of polybrene. Forty‐eight hours later, GC cells were cultured in a medium containing puromycin to select stable clones. The cells were sorted into the following categories: Mock groups represented cells without any treatment; BGC‐823‐Ctrl or NCI‐N87‐Ctrl, infected with the control Lenti‐shRNA; BGC‐823‐sh‐HOXA10 or NCI‐N87‐sh‐HOXA10, infected with the HOXA10 Lenti‐shRNA; AGS‐Ctrl or HGC‐27‐Ctrl, infected with the lentivirus containing the control fragment; AGS‐HOXA10‐OV or HGC‐27‐HOXA10‐OV, infected with the lentivirus containing the HOXA10 fragment. The HOXA10 stably knockdown or overexpression clones were verified by western blot and qRT‐PCR.

### 2.5 BCL2‐overexpressing plasmid and BCL2 selective inhibitor ABT‐199

The BCL2‐overexpressing plasmid was obtained from Hanbio Biotechnology (Shanghai, China). BGC‐823‐sh‐HOXA10+BCL2 or NCI‐N87‐sh‐HOXA10+BCL2 represented BGC‐823‐sh‐HOXA10 or NCI‐N87‐sh‐HOXA10 cells transfected with BCL2‐overexpressing plasmid using Lipofectamine 2000 (Invitrogen).

The BCL2 selective inhibitor ABT‐199 was obtained from MedChem Express. AGS‐HOXA10‐OV+ABT‐199 or HGC‐27‐HOXA10‐OV+ABT‐199 indicated AGS‐HOXA10‐OV or HGC‐27‐HOXA10‐OV cells treated with ABT‐199.

### 2.6 Cell counting kit‐8 assay

The cell counting kit‐8 (CCK‐8) (Dojindo) assay was employed to generate cell growth curves and evaluate cell proliferation. Cells were plated in 96‐well cell culture plates at a density of 1000 cells/well. At various time points (0, 24, 48, 72 hours), the cells were incubated with 10 μL of CCK‐8 solution for 2 hours at 37°C, and then the absorbance at 450 nm was measured on a Gen5 microplate reader (BioTek). The experiment was performed independently in triplicate.

### 2.7 Colony formation assay

To evaluate the colony formation ability, 500 log‐phase cells were seeded in 6‐cm culture dishes. After a 14‐day incubation, the cells were fixed in methyl alcohol for 15 minutes and stained with crystal violet for 15 minutes. Then, the colonies were counted. The experiment was performed independently in triplicate.

### 2.8 Cell apoptosis assay

The cell apoptosis assay was performed with the Annexin V/PI apoptosis detection kit (Yeasen). After collecting and resuspending, the cells were stained with Annexin V and propidium iodide (PI) for 15 minutes in the dark and then analyzed by flow cytometry (BD Accuri). The experiment was performed independently in triplicate.

### 2.9 Nude mice xenograft tumor formation assay

Four‐week male athymic BALB/c nude mice were purchased from Shanghai Laboratory Animal Center, CAS (SLACCAS), 5 × 10^6^ cells were subcutaneously injected into the nude mice. For in vivo imaging assay, AGS cells labeling firefly luciferase were injected into the nude mice. The mice were euthanized 21 days later. Tumors were isolated, and then the weight, width, and length were measured. Tumor volume was calculated using the following formula: volume = width^2^ × length × 0.5. This study was carried out in strict accordance with the Guide for the Care and Use of Laboratory Animals of the National Institutes of Health.

### 2.10 Bioinformatics analysis of HOXA10 and BCL2

The analysis was designed to comprehensively evaluate the relationship between HOXA10 and BCL2. First of all, we uploaded official gene symbols of “HOXA10” and “BCL2” to NetworkAnalyst (http://www.networkanalyst.ca/) and proceeded a “TF‐gene interactions” analysis.^16^ Then we uploaded gene names “HOXA10” and “BCL2” of Homo sapiens to the Search Tool for the Retrieval of Interacting Genes (STRING) (https://string-db.org/) and generated the protein‐protein interaction (PPI) networks between them.^17^ We intended to search potential binding positions of HOXA10 in the BCL2 promoter region by uploading BCL2 promoter sequence to JASPAR (http://jaspar.genereg.net/).^18^

### 2.11 Chromatin immunoprecipitation and quantitative PCR

Chromatin immunoprecipitation (ChIP) was performed in BGC‐823 cells. In brief, BGC‐823 cells (4 × 10^6^ per ChIP) in 15‐cm culture dishes were cross‐linked with 1% formaldehyde and quenched by glycine. Cells were then lysed and nuclei were treated with micrococcal nuclease for 20 minutes at 37°C. The reaction was stopped with 0.5 mol/L EDTA, samples were then sonicated to disrupt nuclear membrane. After centrifugation, the supernatant was collected which contained the chromatin. Chromatin solutions were, respectively, incubated with antibodies anti‐HOXA10 (Santa Cruz, USA), anti‐Histone H3, and anti‐normal rabbit IgG (CST, USA). And then, they were rotated at 4°C overnight, followed by incubation at 4°C with ChIP‐grade protein G magnetic beads. Next, the beads were washed. The cross‐links were reversed at 65°C for 2 hours, and DNA was purified and used for ChIP‐qPCR analysis. For the ChIP‐qPCR experiments, a set of primers was designed against BCL2 promoter region and the primers’ sequence was listed below:

Primer 1‐F: TATGTGAGAGAAGTTGGCTTG; Primer 1‐R: AGATGGCTTTTGCTATGTTG； Primer 2‐F: GCGGATAACGAGGTCAGGA; Primer 2‐R: ACGCCACTACGCTCTGCTAAT； Primer 3‐F: CCCTATTAAGTAAGCCGCTGTG; Primer 3‐R: GTACGCGCAAGCAGACAGT； Primer 4‐F: CTACGAGGCAAAGGTGGAG; Primer 4‐R: GGTTTATCAAGGGCTTTACGAC. For the detection of ChIP‐generated DNA, real‐time PCR was performed as described above. The DNA of PCR products was qualified by 3% agarose gel electrophoresis and then photographed under ultraviolet ray.

### 2.12 Immunohistochemistry

The tissue sections were deparaffinized in xylene and rehydrated in a graded series of ethanol followed by epitope retrieval in citrate buffer (pH = 6.0). HOXA10, BCL2, and Ki67 expressions were detected using antibodies anti‐HOXA10 (1:100, Santa Cruz, USA), anti‐BCL2 (1:100, CST, USA), and anti‐Ki67 (1:600; GeneTex), respectively. After the secondary antibody incubation with Envision System Plus‐HRP, the slides were rinsed and counterstained with Mayer's hematoxylin. Finally, sections were imaged and evaluated.

### 2.13 Statistical analysis

All data were shown as mean ± SD. The significance of the differences between groups was determined using the two‐tailed Student's *t* test or one‐way ANOVA. Statistical significance (*P* value) was calculated by SPSS 22.0 software (SPSS). For all tests, *P* value < .05 was considered statistically significant.

## 3 RESULTS

### 3.1 HOXA10 expression was upregulated in GC tissues

**Figure 1:**
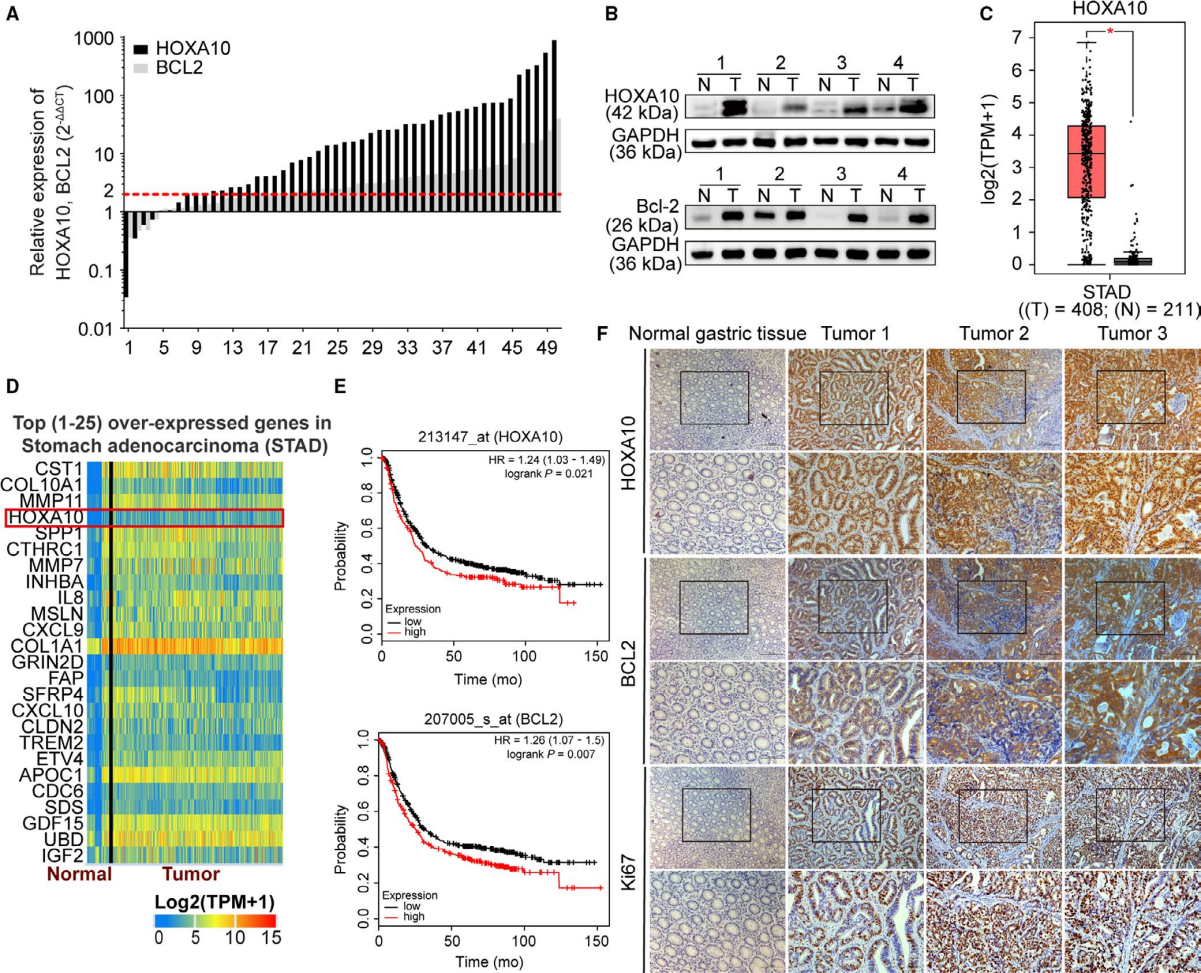
HOXA10 and BCL2 expressions were elevated in GC tissues. A and B, qRT‐PCR and western blot indicated that the expression of HOXA10 was higher in GC tissues (qRT‐PCR values were arranged from low to high). C, HOXA10 was elevated in the STAD reanalyzed from GEPIA. D, HOXA10 ranked fourth of the top 25 overexpressed genes in the STAD from UALCAN. E, Higher expression of HOXA10, BCL2 demonstrated lower overall survival rate in GC patients reanalyzed from the Kaplan‐Meier Plotter. F, Representative immunohistochemical staining showed that HOXA10, BCL2, and Ki67 were upregulated in GC tissues. Original magnification, 100× (200× for insert images). GC, gastric cancer; qRT‐PCR, quantitative real‐time polymerase chain reaction; STAD, stomach adenocarcinoma dataset

Quantitative real‐time polymerase chain reaction (qRT‐PCR) showed that HOXA10 mRNA expression level was markedly upregulated in 50 paired GC samples (Figure 1A) (43/50, 86%). Western blot indicated that HOXA10 had a higher protein level in GC tissues than normal gastric mucosae (Figure 1B). Using bioinformatics analysis, we found that HOXA10 was markedly upregulated in GC tissues and ranked fourth of the top 25 overexpressed genes in the stomach adenocarcinoma dataset reanalyzed from the database GEPIA^19^ and UALCAN^20^ (http://gepia.cancer-pku.cn/detail.php?gene=HOXA10 and http://ualcan.path.uab.edu/cgi-bin/TCGAExHeatMap2.pl?size=25&cancer=STAD) (Figure 1C,D). Besides, the database Kaplan‐Meier Plotter (http://kmplot.com/analysis/) indicated that GC patients with higher HOXA10 (Affymetrix probe set ID 213147_at) expression had worse prognosis (Figure 1E).^21^ Immunohistochemistry assay demonstrated that HOXA10 was positively stained in GC specimens (Figure 1F). Altogether, HOXA10 expression was elevated at both the mRNA and protein levels.

### 3.2 HOXA10 promoted proliferation and repressed apoptosis in GC cells

To further investigate the functional role of HOXA10 and select appropriate cell lines for knockdown or overexpression in GC cells, qRT‐PCR and western blot were conducted to detect HOXA10 expression level in the following GC cell lines: BGC‐823, HGC‐27, SGC‐7901, AGS, NCI‐N87, KATO‐III, MGC‐803, and normal gastric mucosae cell line GES‐1. Notably, HOXA10 expression level in BGC‐823 and NCI‐N87 cells was relatively higher, while the expression level in AGS and HGC‐27 cells was less abundant (Figure S1A,B). To further confirm the tumor formation ability of these GC cell lines, they were injected into nude mice. And, the expression level of HOXA10 was verified by western blot and immunohistochemistry (Figure S1C‐E). Therefore, we employed HOXA10 Lenti‐shRNA to suppress HOXA10 expression in BGC‐823 and NCI‐N87 cells, and adopted lentivirus containing the HOXA10 fragment to elevate HOXA10 expression in AGS and HGC‐27 cells. The efficiency of HOXA10 knockdown or overexpression was confirmed by western blot and qRT‐PCR (Figure S1F).

**Figure 2:**
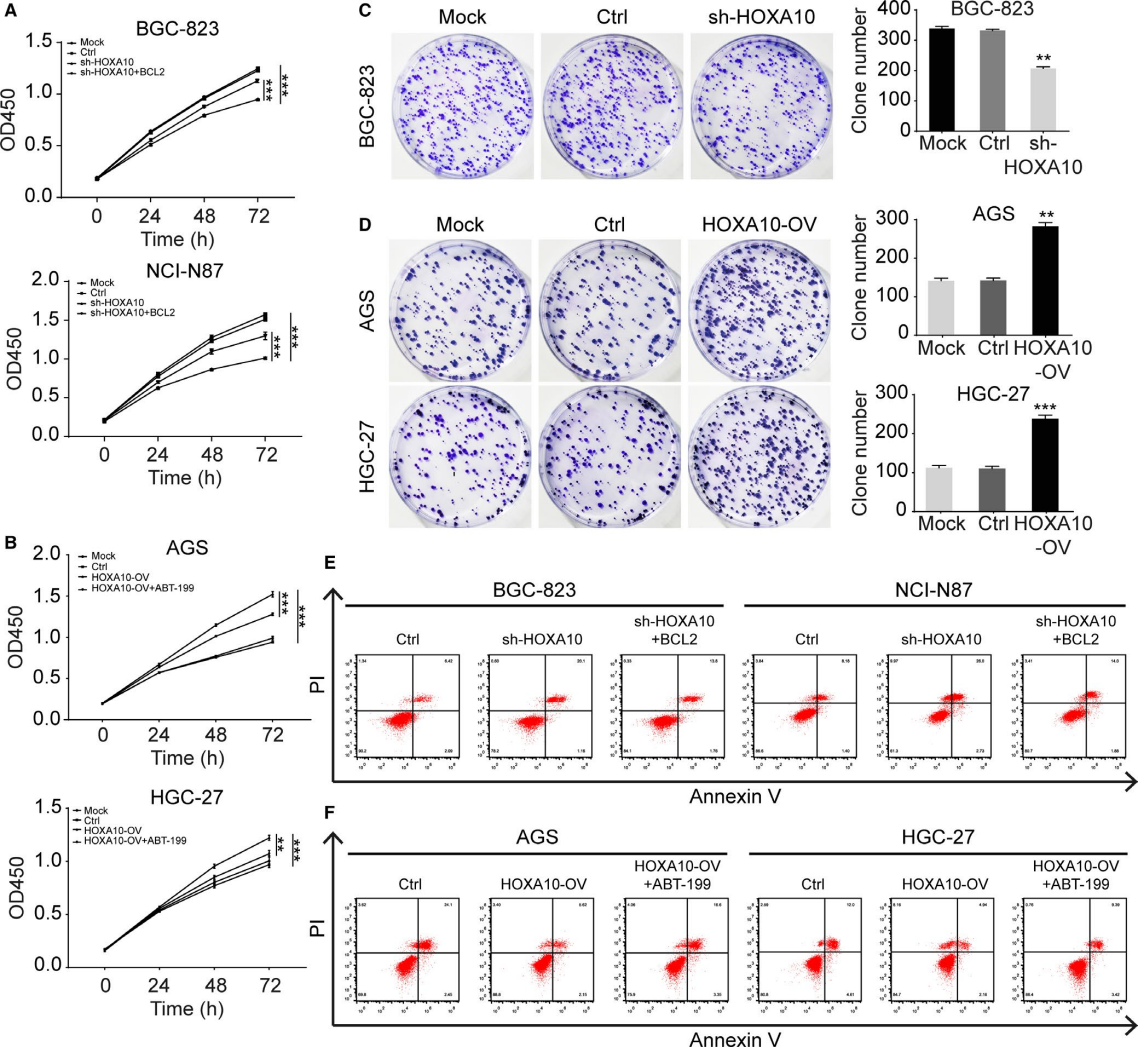
HOXA10 promoted proliferation and repressed apoptosis in GC cells. A and B, The CCK‐8 assay showed that HOXA10 knockdown inhibited cell growth but could be partly rescued by transfecting with BCL2‐overexpressing plasmid. And, HOXA10 overexpression promoted cell growth but could be partially impaired with treatment of BCL2 selective inhibitor ABT‐199. C and D, HOXA10 knockdown inhibited colony formation in BGC‐823 cells while HOXA10 overexpression enhanced colony formation in AGS and HGC‐27 cells. E and F, The cell apoptosis assay showed the percentage of apoptotic cells was higher in HOXA10‐knockdown cells (BGC‐823‐sh‐HOXA10, NCI‐N87‐sh‐HOXA10) compared with the control cells, but could be partly reduced by transfecting with BCL2‐overexpressing plasmid. And, the portion of apoptotic cells was lower in HOXA10‐overexpressinig cells (AGS‐HOXA10‐OV, HGC‐27‐HOXA10‐OV) compared with the corresponding control cells, but could be partially rescued with treatment of ABT‐199. **P < .01, ***P < .001. CCK‐8, cell counting kit‐8. GC, gastric cancer

To study the effects of HOXA10 on GC cell proliferation, the CCK‐8 and colony formation assays were performed. We examined the absorbance at 450 nm to determine cell growth ability at time points of 0, 24, 48, 72 hours. The growth curves indicated that HOXA10 knockdown suppressed the proliferation of BGC‐823 and NCI‐N87 cells (Figure 2A). On the contrary, cell growth was promoted in AGS and HGC‐27 cells with HOXA10 overexpression (Figure 2B). Besides, colony formation assays displayed HOXA10 knockdown decreased the colony numbers of BGC‐823 cells (Figure 2C) while overexpressed HOXA10 produced a nearly twofold increase of the colony numbers for AGS and HGC‐27 cells (Figure 2D). Hence, HOXA10 knockdown impaired the proliferation ability of GC cells while HOXA10 overexpression strengthened that ability.

Additionally, we performed flow cytometry to detect GC cells apoptosis and found that HOXA10‐knockdown cells (BGC‐823‐sh‐HOXA10, NCI‐N87‐sh‐HOXA10) displayed higher apoptotic cell ratio compared to the control cells, respectively. Complementally, HOXA10‐overexpressing cells (AGS‐HOXA10‐OV, HGC‐27‐HOXA10‐OV) showed lower apoptosis ratio compared to the respective control cells (Figure 2E,F and Figure S3). These data indicated that HOXA10 might promote GC cell proliferation by inhibiting apoptosis.

### 3.3 HOXA10 promoted tumor growth in the nude mice xenograft tumor formation assay

**Figure 3:**
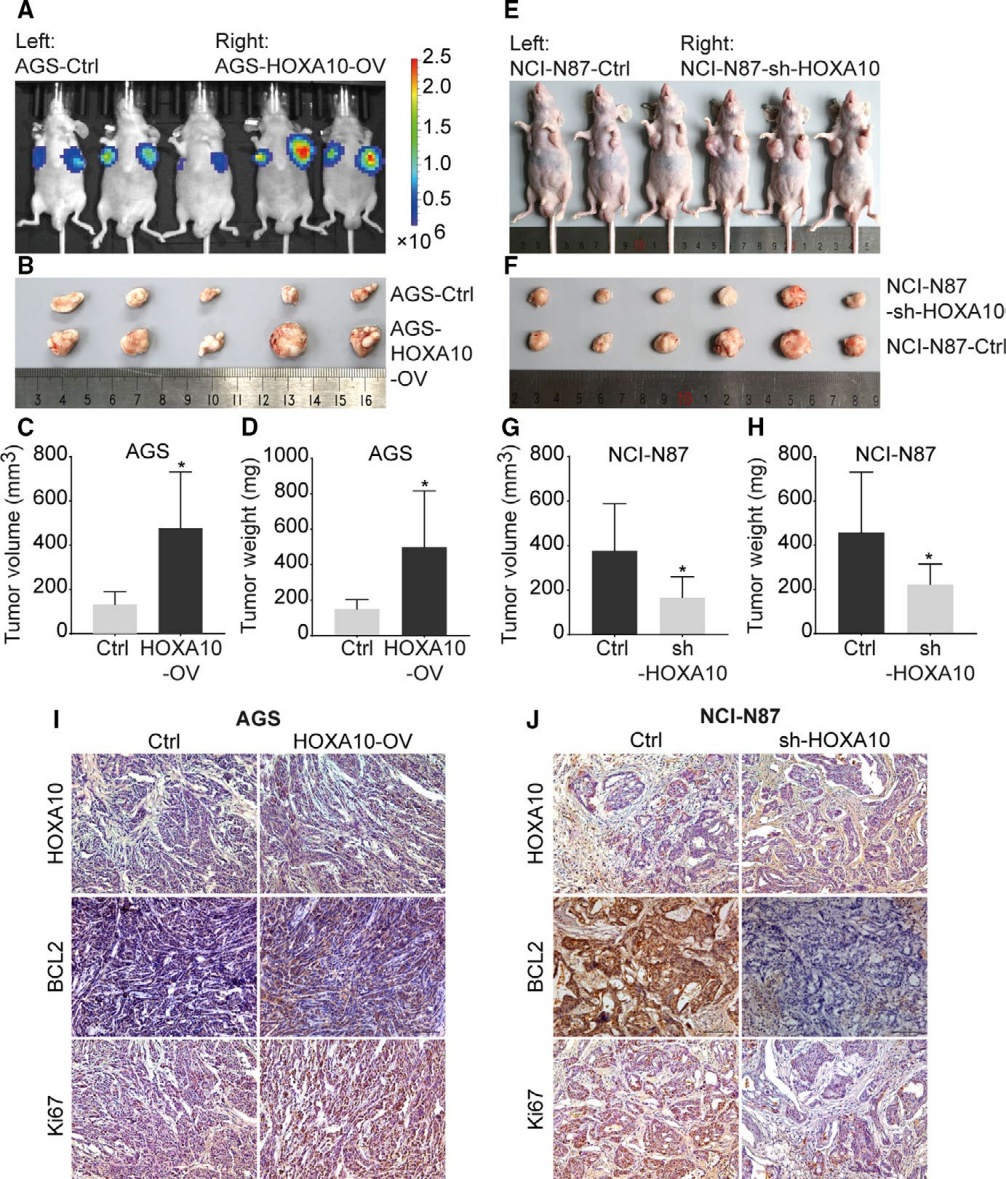
HOXA10 promoted tumor growth in the nude mice xenograft tumor formation assay. A, Tumors formed by AGS‐HOXA10‐OV cells demonstrated an elevation of bioluminescent signal. B‐D, The weight and volume of tumors formed by AGS cells in different groups. HOXA10 overexpression promoted xenograft tumor growth in nude mice. E‐H, The weight and volume of tumors formed by NCI‐N87 cells showed HOXA10 knockdown inhibited xenograft tumor growth in nude mice. I and J, Representative immunohistochemical staining of HOXA10, BCL2, and Ki67 in xenograft tumors. Original magnification, 200×. *P < .05

To testify the effect of HOXA10 on tumor growth in vivo, cell lines with altered expression of HOXA10 were injected into nude mice. AGS‐HOXA10‐OV cells formed significantly larger tumors compared with AGS‐Ctrl cells, as evaluated by tumor volume, weight, and bioluminescence (Figure 3A‐D). In contrast, NCI‐N87‐sh‐HOXA10 cells resulted in a significant reduction of the tumor volume and weight compared with NCI‐N87‐Ctrl cells (Figure 3E‐H). These results suggested that HOXA10 overexpression could enhance tumor growth while HOXA10 knockdown could deteriorate tumor growth in vivo. Moreover, immunohistochemical staining showed that HOXA10 was upregulated in tumors formed by AGS‐HOXA10‐OV cells while weakened in tumors formed by NCI‐N87‐sh‐HOXA10 cells (Figure 3I,J). Altogether, these findings demonstrated that HOXA10 enhanced GC cells tumor growth in vivo.

### 3.4 Elevated HOXA10 upregulated BCL2 expression via binding to its promoter region

**Figure 4:**
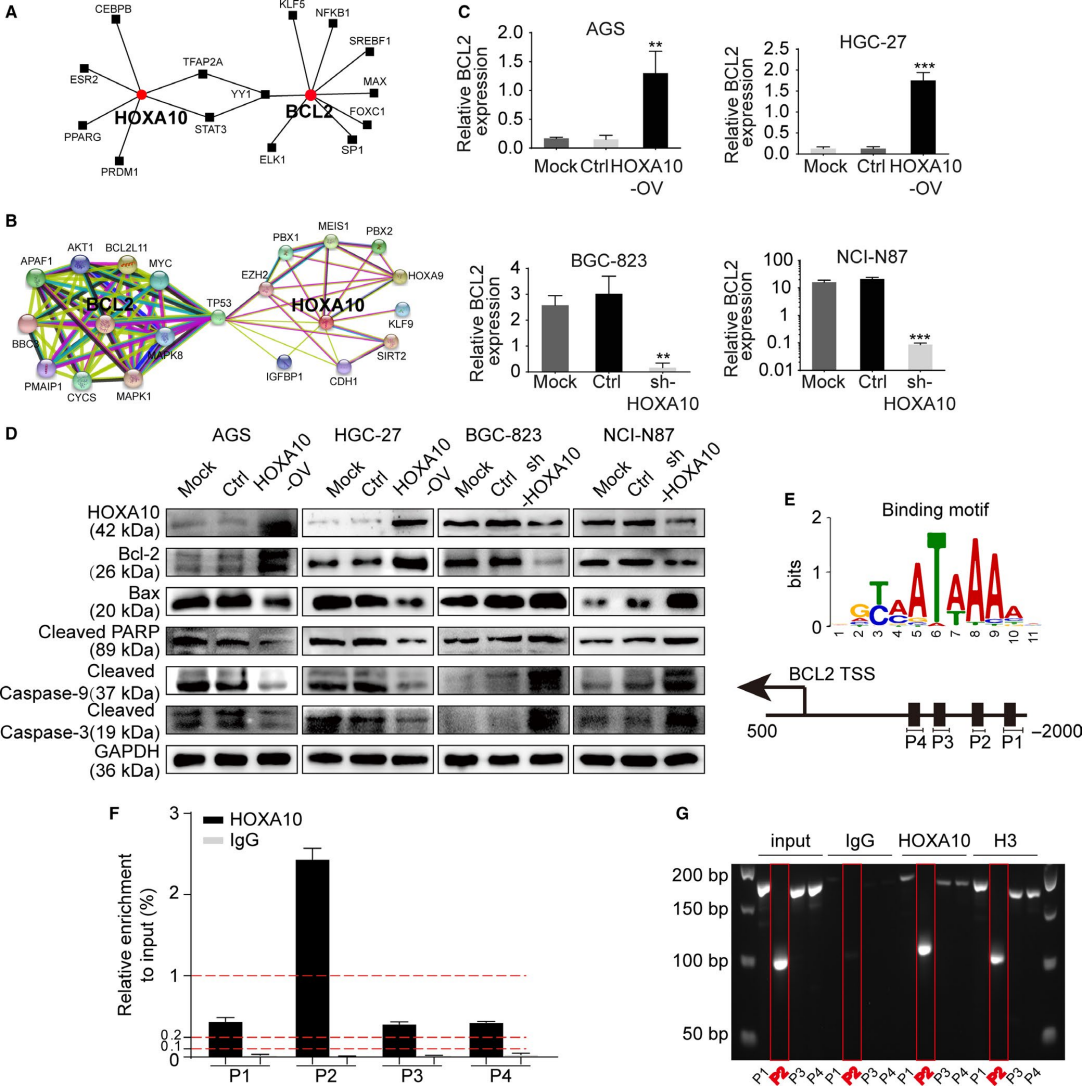
The bioinformatics analysis, qRT‐PCR, western blot, and ChIP‐qPCR assay revealed that HOXA10 might induce BCL2 expression via binding to its promoter region. A, The “TF‐gene interactions” analysis of HOXA10 and BCL2 in the database NetworkAnalyst. B, A PPI network was built between HOXA10 and BCL2 with the database STRING. C, qRT‐PCR showed the relative BCL2 mRNA level in different cells with changed HOXA10 expression. D, Expression of BCL2, Bax, cleaved forms of Caspase‐9, Caspase‐3, and PARP in different GC cells with altered HOXA10 expression. E, HOXA10 binding motif acquired from JASPAR and the relative primer position within the BCL2 promoter region. F, ChIP‐qPCR assay indicated the possible positive HOXA10 binding sites across the BCL2 promoter region. G, 3% agarose gel electrophoresis of ChIP‐qPCR products. **P < .01, ***P < .001. ChIP‐qPCR, chromatin immunoprecipitation and quantitative PCR; PPI, protein‐protein interaction; qRT‐PCR, quantitative real‐time polymerase chain reaction; TF‐gene, transcription factor‐gene

By bioinformatics analysis, we discovered that HOXA10 could regulate BCL2 after a “TF‐gene interactions” analysis in the database NetworkAnalyst (Figure 4A). Then a PPI network was built between HOXA10 and BCL2 with the database STRING (Figure 4B).

Subsequently, we performed qRT‐PCR experiment and found the mRNA level of BCL2 was markedly higher in HOXA10‐overexpressing cells while significantly lower in HOXA10‐knockdown cells (Figure 4C). Consistent with the qRT‐PCR results, the expression of anti‐apoptosis protein BCL2 was significantly upregulated in the HOXA10‐overexpressing cells while the proapoptotic protein Bax was impaired, thus decreased the Bax/BCL2 ratio. Meanwhile, the cleaved forms of Caspase‐9, Caspase‐3, and PARP were all reduced, respectively. Furthermore, in the HOXA10‐knockdown cells, we observed the opposite alteration (Figure 4D).

To further study if there existed the transcriptional regulation relationship between HOXA10 and BCL2, we uploaded BCL2 promoter sequence to JASPAR and found potential binding positions of HOXA10. Then, we designed four primers targeting different promoter regions of BCL2 according to the HOXA10 binding motif and then performed the ChIP‐qPCR assay. As a result, HOXA10‐ChIP group had a significant enrichment in primer 2 compared with other primers, which was normalized to input. The DNA of ChIP‐qPCR products was used to run a 3% agarose gel electrophoresis and the result verified again that HOXA10‐ChIP group bound most to the primer 2 within the BCL2 promoter region (Figure 4E‐G).

### 3.5 HOXA10‐induced phenotypes were partly rescued with BCL2 selective inhibitor ABT‐199

To further detect if HOXA10 might exert anti‐apoptosis function by upregulating BCL2, we performed the rescue experiments. The CCK‐8 assay and cell apoptosis assay demonstrated the effects of rescue experiments. The CCK‐8 assay showed that the proliferation ability was partly impaired in HOXA10‐overexpressing cells with treatment of ABT‐199. And, the cell proliferation phenotype was partially reversed in HOXA10‐knockdown cells after transfecting with BCL2‐overexpressing plasmid (Figure 2A,B).

The cell apoptosis assay indicated that after treating the HOXA10‐overexpressing cells with ABT‐199, the ratio of apoptotic cells was partly elevated. The cell apoptosis ratio of AGS‐HOXA10‐OV+ABT‐199 cells was higher than the ratio of AGS‐HOXA10‐OV cells. The apoptotic ratio of HGC‐27‐HOXA10‐OV+ABT‐199 cells was higher than that of HGC‐27‐HOXA10‐OV. And in the HOXA10‐knockdown cells, BCL2‐overexpressing plasmid also demonstrated the rescued effects (Figure 2E,F and Figure S3).

Besides, western blot experiment indicated the rescued effects as well. We found that after treatment with ABT‐199 in the HOXA10‐overexpressing cells, the expression of BCL2 was suppressed while cleaved Caspase‐3 and cleaved PARP were elevated. And in the HOXA10‐knockdown cells, we observed the opposite protein expression change of BCL2, cleaved Caspase‐3, and cleaved PARP after transfecting with BCL2‐overexpressing plasmid (Figure S2).

And then, we performed the immunohistochemistry assay in the xenograft tumors. It showed the expression of BCL2 was significantly higher in tumors formed by AGS‐HOXA10‐OV cells. Ki67, a cell proliferation indicator, also demonstrated a stronger staining in AGS‐HOXA10‐OV cells (Figure 3I,J).

### 3.6 BCL2 expression was elevated in GC tissues and predicted poor prognosis

We then detected the expression of BCL2 in GC tissues. qRT‐PCR showed that BCL2 mRNA expression level was upregulated in GC samples (Figure 1A) (34/50, 68%). Western blot revealed a significant elevation of BCL2 protein expression in GC tissues than normal mucosae (Figure 1B). Besides, immunohistochemical staining demonstrated that HOXA10, BCL2, and Ki67 were upregulated in GC tissues (Figure 1F). Furthermore, the database Kaplan‐Meier Plotter indicated that higher BCL2 (Affymetrix probe set ID 207005_s_at) expression also predicted worse prognosis in GC patients (Figure 1E).^21^

## 4 DISCUSSION

Much scientific evidence indicated that the expression of genes responsible for normal embryo development was aberrant and contributed to carcinogenesis.^22^, ^23^, ^24^ In the past years, HOX genes emerged as transcriptional regulators responsible for embryogenesis and were involved in the development of various cancers.^4^ As an essential part of HOXA family genes, HOXA10 expression was crucial for ensuring normal development and differentiation of hematopoietic stem cells.^25^ Moreover, emerging evidence indicated that upregulation of HOXA10 was associated with different cancer types.^26^, ^27^

In our present study, we demonstrated that HOXA10 was elevated in GC tissues. A series of in vitro and in vivo experiments suggested that upregulated HOXA10 facilitated GC cell proliferation. Besides, overexpressed HOXA10 decreased the portion of apoptotic cells and inhibited intrinsic apoptosis pathway, shown as elevated expression of BCL2 and declined expression of Bax, cleaved forms of Caspase‐9, Caspase‐3, and PARP.

Cell apoptosis is closely connected with cell proliferation and exerts important roles in human development.^28^ BCL2 family proteins play indispensable roles in intrinsic (mitochondrial) apoptosis pathway by modulating apoptotic threshold, and their dysregulation facilitates tumorigenesis.^29^ Notably, Tang et al identified that miR‐135a targeted HOXA10 3’‐UTR directly repressed HOXA10 expression, BCL2 expression, and resulted in cell apoptosis with concomitant enhancement of Caspase‐3 and p53.^30^ However, the underlying mechanism between HOXA10 and BCL2 was not elucidated.

In this study, we made an in‐depth investigation to explore if there remained regulatory relationship between HOXA10 and BCL2 in GC. To start with, bioinformatics analysis based on the database listed above revealed that HOXA10 might regulate BCL2 expression. Through qRT‐PCR and western blot, we observed the expression of BCL2 was significantly higher in HOXA10‐overexpressing cells compared with the control cells, and the expression of BCL2 was dramatically lower in HOXA10‐knockdown cells than that in the control cells. These results demonstrated that HOXA10 might regulate the expression of BCL2. To further elucidate the regulatory mechanism between transcription factor HOXA10 and BCL2, ChIP‐qPCR assay was performed. As a result, we found that HOXA10 might bind to the BCL2 promoter region and thus induce its expression.

Subsequently, we performed the rescue experiment with BCL2‐overexpressing plasmid and BCL2 selective inhibitor ABT‐199. The CCK‐8 assay showed the proliferation ability was enhanced in HOXA10‐knockdown cells after transfecting with BCL2‐overexpressing plasmid, and the proliferation ability was partly impaired in HOXA10‐overexpressing cells with treatment of ABT‐199.

Besides, the cell apoptosis assay indicated that after treating the HOXA10‐overexpressing cells with ABT‐199, the ratio of apoptotic cells was elevated. And in the HOXA10‐knockdown cells, the BCL2‐overexpressing plasmid also demonstrated the rescued effects (Figure 2E,F and Figure S3). The experiments above indicated that HOXA10 might exert anti‐apoptosis function by upregulating BCL2. Significantly, there might exist some other pathways explaining the phenotype that HOXA10 promoted GC cell proliferation and repressed apoptosis. Several other downstream targets of HOXA10, such as p53 and p21,^31^, ^32^ were observed to play multiple roles in cell proliferation and apoptosis.

Thereafter, we detected the expression of BCL2 in GC tissues. qRT‐PCR and western blot revealed that BCL2 was elevated in GC tissues. And, the representative immunohistochemical staining showed that HOXA10, BCL2, and Ki67 were upregulated in GC tissues. Besides, the database Kaplan‐Meier Plotter indicated higher expression of HOXA10, BCL2 predicted poor prognosis in GC patients. It is noteworthy that BCL2 inhibitors, such as venetoclax (ABT‐199), are clinically explored in several cancer types.^33^, ^34^ However, studies have indicated that the expression levels of anti‐apoptotic proteins, BCL2, Bcl‐xL, and Mcl‐1, are highly variable, manifesting various disease prognosis and venetoclax sensitivity.^34^, ^35^, ^36^ Thus, further studies are needed to explore whether BCL2 upstream genes, such as HOXA10, could help develop new therapy strategies for GC.

In summary, we demonstrated that expression of HOXA10 and BCL2 was significantly upregulated in GC tissues. HOXA10 overexpression promoted GC cell proliferation, enhanced tumor growth, and inhibited apoptosis. Through bioinformatics analysis, western blot, ChIP‐qPCR, and the rescue experiment, we found that HOXA10 might upregulate the expression of BCL2 via binding to the BCL2 promoter region. All these findings suggested that HOXA10 might promote GC cell proliferation by upregulating BCL2 expression and inhibiting apoptosis.

## CONFLICT OF INTEREST

The authors have no conflict of interest to declare**.**

## AUTHORS CONTRIBUTION

CZZ and CLS designed and coordinated the study, drafted and revised the manuscript. CLS and YH carried out cell culture, animal experiments, and related experiments. CLS and HL carried out the statistical analysis. ZWQ, ZQC, and YL helped collect tissue samples. SL and HMS evaluated the immunohistochemistry staining. All authors read and approved the final manuscript.
