# Supplementary figures and images for: HOXA10 induces BCL2 expression, inhibits apoptosis, and promotes cell proliferation in gastric cancer
Source: Cancer Med. 2019 Jul 30;8(12):5651–61. doi: 10.1002/cam4.2440 (PMC6745829; doi:10.1002/cam4.2440)

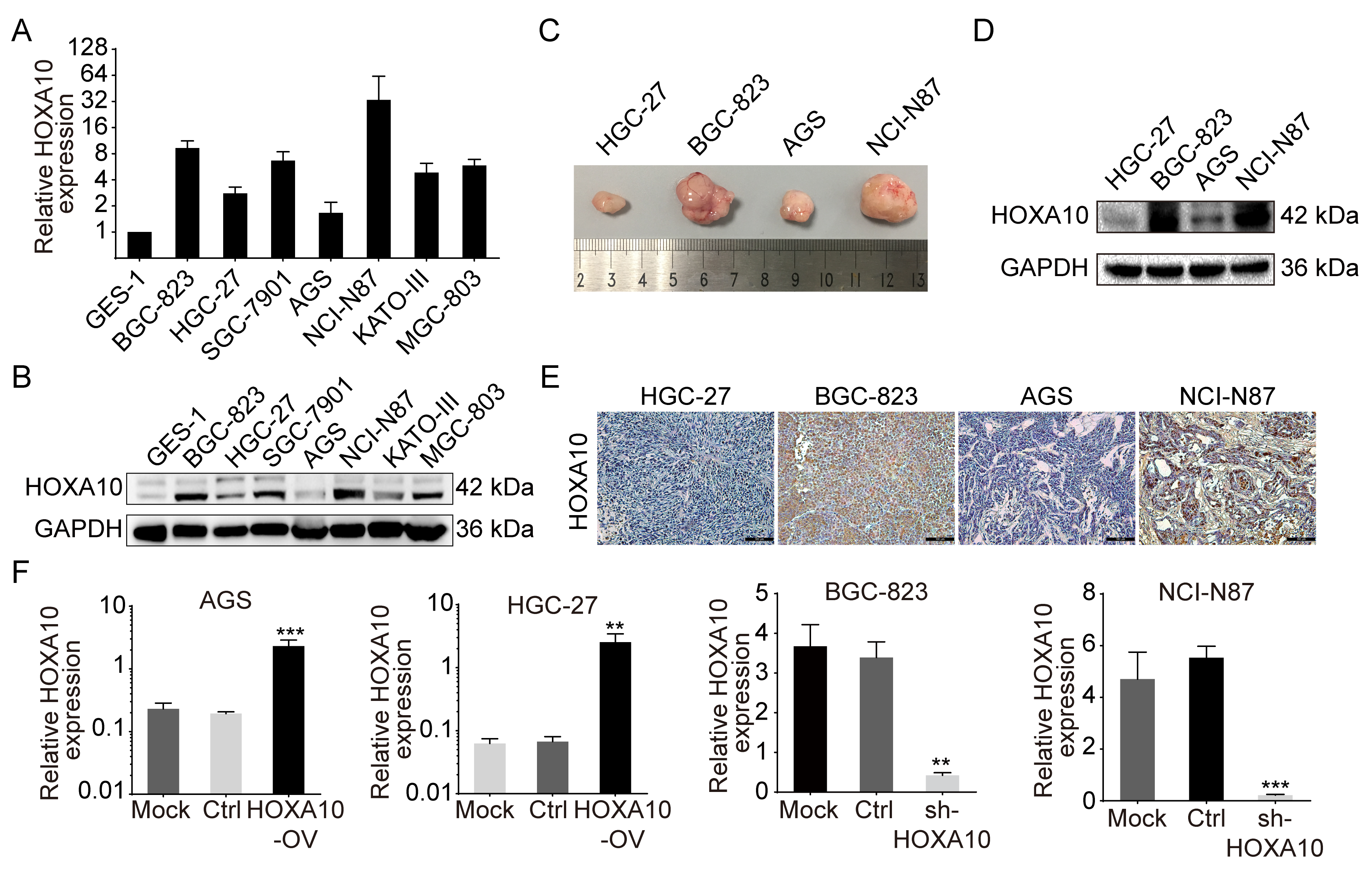

Supplement: Supplementary file 1 [file CAM4-8-5651-s001.tif]

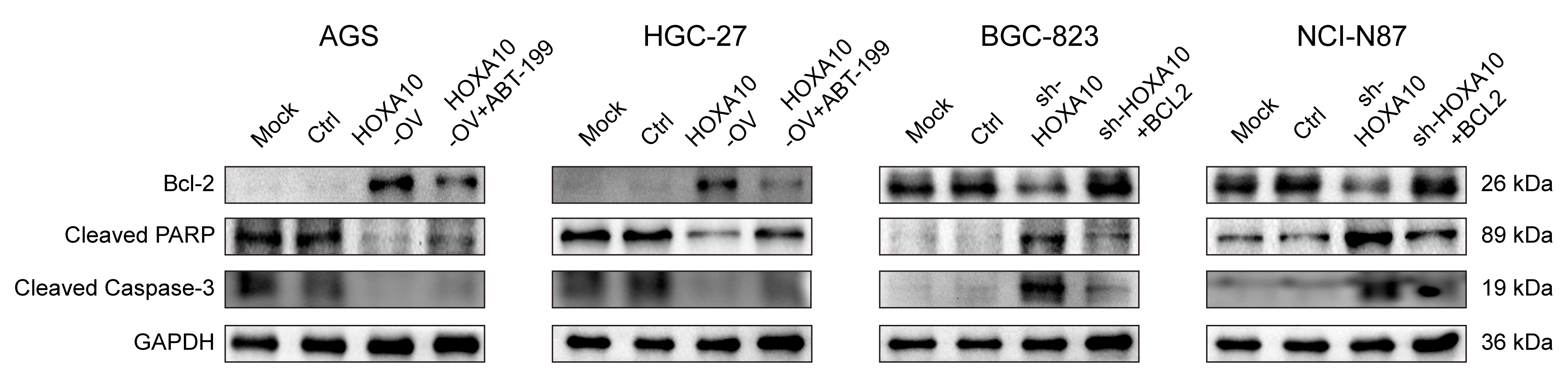

Supplement: Supplementary file 2 [file CAM4-8-5651-s002.tif]

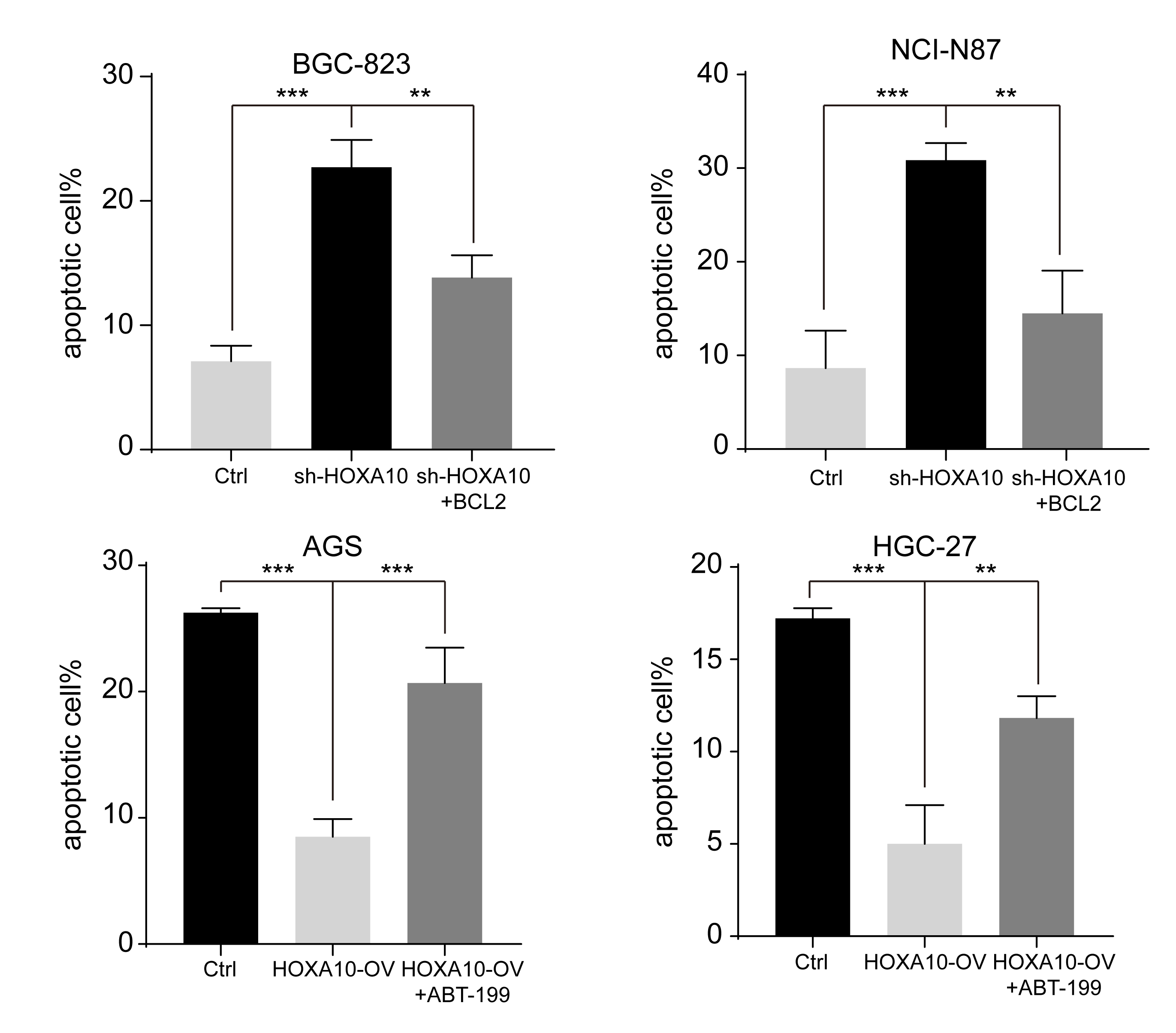

Supplement: Supplementary file 3 [file CAM4-8-5651-s003.tif]
